# Exploring the Potential Role of the Gut Microbiome in Chemotherapy-Induced Neurocognitive Disorders and Cardiovascular Toxicity

**DOI:** 10.3390/cancers13040782

**Published:** 2021-02-13

**Authors:** Sona Ciernikova, Michal Mego, Michal Chovanec

**Affiliations:** 1 Department of Genetics, Cancer Research Institute, Biomedical Research Center of the Slovak Academy of Sciences, 845 05 Bratislava, Slovakia; 2 2nd Department of Oncology, Faculty of Medicine, Comenius University, Bratislava and National Cancer Institute, 833 10 Bratislava, Slovakia; misomego@gmail.com (M.M.); michal.chovanec1@gmail.com (M.C.)

**Keywords:** microbiome, cancer survivors, chemotherapy-induced side effects, cognitive impairment, microbiota–gut–brain axis, cardiovascular toxicity, microbiota modulation

## Abstract

**Simple Summary:**

While lifesaving achievements have allowed cancer to be cured in many patients, survivors cured of cancer may suffer from long-term adverse treatment sequelae, substantially altering their quality of life and reintegration into normal life. Increasing evidence suggests the emerging role of the microbiome in chemotherapy-induced late effects affecting cognitive functions and the cardiovascular system. Moreover, existing data from animal models and patients with neurocognitive disorders and cardiovascular diseases outline the possibility that microbiota modulation might potentially prevent or mitigate the psycho-physiological deficits following chemotherapy and help to improve the behavioral comorbidities, cognitive functions, and quality of life in cancer survivors.

**Abstract:**

Chemotherapy, targeting not only malignant but also healthy cells, causes many undesirable side effects in cancer patients. Due to this fact, long-term cancer survivors often suffer from late effects, including cognitive impairment and cardiovascular toxicity. Chemotherapy damages the intestinal mucosa and heavily disrupts the gut ecosystem, leading to gastrointestinal toxicity. Animal models and clinical studies have revealed the associations between intestinal dysbiosis and depression, anxiety, pain, impaired cognitive functions, and cardiovascular diseases. Recently, a possible link between chemotherapy-induced gut microbiota disruption and late effects in cancer survivors has been proposed. In this review, we summarize the current understanding of preclinical and clinical findings regarding the emerging role of the microbiome and the microbiota–gut–brain axis in chemotherapy-related late effects affecting the central nervous system (CNS) and heart functions. Importantly, we provide an overview of clinical trials evaluating the relationship between the gut microbiome and cancer survivorship. Moreover, the beneficial effects of probiotics in experimental models and non-cancer patients with neurocognitive disorders and cardiovascular diseases as well as several studies on microbiota modulations via probiotics or fecal microbiota transplantation in cancer patients are discussed.

## 1. Introduction

Modern oncology has produced substantial advancements in cancer treatment, prolonging the lives of patients. Therefore, the population of long-term survivors is continuously increasing. A report from the American Cancer Society estimated that the number of all cancer survivors in the United States will exceed 20 million by 2026, resulting in almost double the number reported in 2012. Survivors will then represent 4% of the overall population, with a further expected rise in future years [1,2]. Cancer survivors face the long-term side effects of cancer treatment, with a number of late complications increasing the risk of cardiovascular diseases, second malignant neoplasms, the impairment of fertility, peripheral neuropathy, renal toxicity, and several other chemotherapy-related delayed toxicities [3,4]. Moreover, discoveries and updates have shown altered neurocognitive functions among cancer survivors during and after treatment with chemotherapy or radiotherapy [5,6,7]. Recent findings have reported that other treatments including hormone therapies and targeted treatments can also contribute to cancer-related cognitive impairment [7]. Additionally, emerging data from preclinical models have reported neuroinflammatory and cognitive consequences of radiotherapy combined with immunotherapy [8]. Multiple candidate mechanisms for these cognitive sequelae have been proposed, including DNA damage induced by the direct or indirect oxidative impact of chemotherapy, gene variations related to neural repair and plasticity, chemotherapy-induced hormonal changes, or immune-mediated proinflammatory mechanisms causing microvascular injury [9,10,11,12,13]. Interestingly, recent evidence from animal models suggests that chemotherapy-induced changes in the intestinal membrane integrity and microbial diversity may be associated with the treatment-related psychoneurological immune-related mechanism of chemotherapy-induced neuroinflammation [14].

Cancer microbiome research represents an emerging field that gradually comes to the fore of clinical oncology from multiple perspectives [15]. Preclinical findings as well as clinical trials have uncovered the crucial role of the human gut microbiome in tumorigenesis, particularly in gastric and colorectal cancers, but also in liver, breast, pancreatic, and lung cancer; lymphoma; and others [16,17,18,19,20]. Recently, the microbial analysis of more than 1500 tumor samples and adjacent normal tissue from breast, lung, ovary, pancreas, melanoma, bone, and brain cancer described the intracellular localized bacteria in both cancer and immune cells. Importantly, each tumor type was characterized by a distinct composition of intratumoral microbiota [21]. Increasing evidence from animal models and clinical studies has highlighted the significant impact of the gut microbiome on the efficacy of cancer therapy, concerning mainly immunotherapeutic and chemotherapeutic treatment modalities [22,23,24,25,26,27].

Nowadays, there is no doubt about the devastating effects of chemotherapy on microbial diversity, leading to acute dysbiosis and severe gastrointestinal toxicities [28,29]. In addition, some recent data link the altered microbiome composition to the late effects of treatment in cancer survivors [30,31,32], and several clinical trials regarding this issue have been already completed or are still ongoing (Table 1).

Here, we provide an overview of current knowledge about the emerging role of the microbiome in cancer treatment, proposing the potential relationship between treatment-related microbial shifts and long-term effects mediated by the microbiota–gut–brain axis. Particularly, the associations between chemotherapy-induced gut microbiota disruption and neurocognitive disorders as well as cardiovascular toxicity will be discussed. Several clinical studies concerning the microbiota modulation in chemotherapy-treated survivors as well as mounting research on mouse models and patients with neurological disorders and cardiovascular diseases outside the cancer field suggest that targeting the gut microbiome might represent a perspective trend for improving the quality of life in cancer survivors. However, randomized clinical trials comprising large cohorts are required and may shed light on the still unexplored relationship between the microbiome and the late toxicity of cancer treatment.

## 2. Treatment-Induced Cardiovascular Toxicity and Neurocognitive Disorders in Long-Term Cancer Survivors

Treatment for cancer results in life-threatening and organ-related late toxicities. Several populations of cancer survivors exist to study the late effects of curative treatments. Among these are survivors treated for malignancies as children, adolescents, and young adults; survivors of testicular germ-cell tumors (GCTs), lymphomas, and leukemias; as well as those treated with adjuvant treatment for various types of solid tumors [4,33,34,35,36,37,38]. Late toxic effects are well-described in GCT survivors who serve as a unique research model in this important field. A cure for GCT is achieved with a multimodal approach including surgery, cisplatin-based chemotherapy, and radiotherapy [39]. An increased risk of myocardial infarction (hazard ratio (HR): 6.3 (95% CI: 2.9–13.9)), cerebrovascular morbidity (HR: 6.0 (95% CI: 2.6–14.1)), and venous thromboembolism (HR 24.7 (95% CI: 14.0–43.6)) was identified in patients shortly after treatment with cisplatin-based chemotherapy. Although it decreased to levels of the general population within one year after treatment, the risk of myocardial infarction and cardiovascular disease-related death increased again 10 years after treatment—HR: 1.4 (CI: 1.0–2.0) and HR: 1.6 (CI: 1.0–2.5), respectively [40]. Other works show a 1.5- to 5.7-fold increase in the risk of cardiovascular disease in patients treated with chemotherapy compared to those treated with orchiectomy only [41,42,43]. Treatment with chemotherapy and additional radiotherapy led to a significant increase in risk of cardiovascular disease after a median of 21 years of follow-up (HR = 5.3; 95% CI: 1.5–18.5) [44].

Radiotherapy-induced cardiotoxicity was described in women treated with older radiation techniques before 1975 with higher radiation dosages [45,46,47]. The addition of radiotherapy to surgical treatment resulted in a HR 1.27 (SE 0.07, 2p = 0.0001) for excess mortality >15 years after treatment in the Early Breast Cancer Trialists’ Collaborative Group meta-analysis [48]. Adjuvant chemotherapy with anthracyclines also resulted in a higher cumulative risk (1.5%) of congestive heart failure 10 years after chemotherapy compared to 0% in patients treated with a non-anthracycline regimen [49]. Based on data from the Surveillance, Epidemiology, and End Result database (SEER), older patients have a higher risk of cardiac events when treated with anthracycline-based adjuvant chemotherapy [50,51].

Large cohorts of survivors have provided important evidence of chemotherapy-induced effects on cognitive functioning, mainly in breast cancer but also in colorectal, ovarian, and testicular cancer and lymphoma [6,52,53,54]. The findings from breast cancer survivors revealed that the patients mostly experienced memory loss and problems with attention, information processing, organization, and decision-making [55,56]. In a cohort of 155 GCT survivors, we have shown that treatment with chemotherapy, radiotherapy, or both modalities was associated with self-reported impairment in several domains of cognitive functioning at a median of 10 years after treatment compared to controls treated with orchiectomy only [6]. A study of 28 GCT survivors described lower cognitive performance and white brain matter changes 14 years after chemotherapy [57]. Evidence of decreased cognitive measures in GCT survivors 2–7 years after chemotherapy was provided also by Amidi et al. [58]. Cognitive complaints were also discovered in 581 patients 6 months after adjuvant chemotherapy for breast cancer compared to age-matched controls [5]. It is estimated that one third of all childhood cancer survivors in the United States suffer from cognitive dysfunction [59]. Chemotherapy-induced peripheral neuropathy (CIPN) is another common adverse late effect in cancer survivors. CIPN can persist to some degree in 20–40% of patients, and the important risk factor is a cumulative dose of cisplatin greater than 300 mg/m^2^ [60,61,62,63]. Measurable levels of serum cisplatin were linked to a 2–4 fold increase in the risk of paresthesia after a median follow-up of 20 years [61]. GCT survivors with a higher grade of CIPN also reported substantial impairments in their long-term quality of life and cognition [64].

## 3. Gut Microbiome, Chemotherapy, and Microbiota-Modulated Efficacy of Cancer Treatment

The gut microbiome plays an essential role in enhancing intestinal homeostasis and mucosal barrier integrity. Moreover, the profound effect of intestinal bacteria on the host immune system has been intensively studied [65]. The gut microbiome represents the collective genetic material within the intestinal microbiota. A comprehensive metagenomic approach enables the study of the human microbiome without the need for further isolation and laboratory cultivation. Interestingly, the microbial composition shows considerable diversity not only within individuals but also between different populations. In this context, the American HMP project (Human Microbiome Project), the European project MetaHIT (METAgenomics of the Human Intestinal Tract), and the Asian project AMP (Asian Microbiome Project) were devoted to determining the healthy bacterial composition [66,67,68]. According to their findings, Firmicutes and Bacteroidetes, followed by Proteobacteria, Actinobacteria, Fusobacteria, and Verrucomicrobia, are the most abundant bacterial phyla in a healthy gut microbiome [69]. Firmicutes represent more than 200 different genera, including *Lactobacillus*, *Bacillus*, *Clostridium*, *Enterococcus*, and *Ruminicoccus*. The predominant representatives of Bacteroidetes are *Bacteroides* spp. and *Prevotella* spp., whereas Actinobacteria are mainly presented with *Bifidobacterium* genus [70].

A healthy gut microbiota composition is characterized by a broad microbial diversity and high colonization resistance. Cancer treatment with common chemotherapeutic drugs results in gut dysbiosis accompanied by a decrease in commensal microbes as *Bifidobacterium* and *Lactobacillus* and an increase in opportunistic pathogens such as *Clostridium difficile* [71]. Treatment-induced mucosal barrier disruption leads to a massive proinflammatory immune response, gastrointestinal toxicity [72], and bacterial translocation, followed by the development of severe bacteremia [73]. Goubet et al. described a disrupted gut barrier integrity and perturbed intestinal homeostasis after the treatment with DNA-alkylating agent cyclophosphamide (CY) [74]. The results from sarcoma mouse models revealed the CY-induced changes in microbiota composition as well as the significant translocation of Gram-positive bacteria into mesenteric lymph nodes and spleens [27]. Importantly, a clinical study comprising 36 pediatric leukemic patients receiving high-dose methotrexate chemotherapy and 36 healthy children reported a significant reduction in *Bifidobacterium*, *Lactobacillus*, and *Escherichia coli* in patients’ fecal samples compared to the controls [75]. Furthermore, a significant decrease in Firmicutes and Actinobacteria and an increase in the abundance of Proteobacteria was observed in 28 adult non-Hodgkin’s lymphoma patients after chemotherapy. In addition, the results showed the association between chemotherapy and gastrointestinal mucositis and also the chemotherapy-related functional imbalances in the gut microbial community, resulting in reduced capacity for nucleotide, energy, and vitamin metabolism [76]. Gastrointestinal mucositis, characterized by painful inflammation and the ulceration of the mucous membranes lining the digestive tract represents very frequent toxicity during chemo- and radiotherapy that is responsible for nonspecific symptoms such as nausea, vomiting, abdominal pain, and diarrhea [77]. A recent microbial analysis of breast tumors from untreated or neoadjuvant chemotherapy-treated patients showed chemotherapy-induced shifts in breast tumor microbiome with a significant increase in *Pseudomonas* spp. Moreover, the elevated abundance of *Brevundimonas* and *Staphylococcus* in patients’ primary tumors was shown to be associated with the development of distant metastasis [78].

Recently, studies focusing on the role of bacterial biomarkers in the prediction of the therapeutic outcome in cancer patients have gained much attention. The breakthrough findings from animal models highlighted the beneficial role of commensals in modulating the efficacy of chemotherapeutic agents. Iida et al. demonstrated the link between intestinal microbiota and the release of reactive oxygen species (ROS) by tumor-infiltrating myeloid cells during platinum-based therapy [26]. Accordingly, Viaud et al. observed that the gut microbiota shapes the CY-elicited anticancer response via the stimulation of a specific subset of “pathogenic” T helper 17 (pTh17) cells and memory Th1 immune responses [27]. Recently, clinical studies comprising patients with metastatic melanoma [24,79] or non-small-cell lung cancer and renal cell carcinoma [25] discovered a profound impact of the gut microbiota on immune checkpoint blockade targeting the coinhibitory receptor/ligand system programmed death-1 (PD-1)/PDL-1. Findings from two distinct cohorts of metastatic melanoma patients have demonstrated that responsiveness to PD-1 therapy is defined by an increased abundance of *Faecalibacterium prausnitzii* [24] or *Bifidobacterium longum* [79], respectively. Furthermore, an increased relative abundance of *Akkermansia muciniphila* was observed in PD-1 responders with lung cancer compared to non-responders [25].

According to the findings, the specific effects of commonly used chemotherapy agents/agent-based regimens on neurocognitive and/or cardiovascular system as well as chemotherapy-induced changes in the gut microbiome have been summarized (Table 2).

## 4. The Role of the Gut Microbiota in Brain Development, Cognitive Functioning, and Neurological Disorders

The gut microbiome is a key performer that interacts with host organisms through the production of several metabolites from endogenous compounds and/or from exogenous substrates. Distinct classes of microbiota-derived metabolites including short-chain fatty acids (SCFA), branched-chain amino acids, bile acids, trimethylamine N-oxide, tryptophan, or indole derivatives are an important part of these interactions and play a role in the pathogenesis of multiple disorders. At the same time, they could serve as potential diagnostic and prognostic biomarkers as well as therapeutic targets for the development of new treatments [92].

The human brain was found to be very sensitive to microbial disharmony, and the impact of altered gut microbiota composition on brain physiology and behavior has been documented. Studies mostly from rodent models showed that the alterations in the microbiota-host relationship affect the enteric nervous system (ENS) and activate the neuroimmune signaling pathways influencing brain development and function [93]. Germ-free (GF) mice or mice treated with broad-spectrum antibiotics showed impaired maturation of brain resident immune microglial cells [94,95]. Erny et al. reported the constitutive activity of the host microbiota on the brain innate immune system, showing that microbiota-derived metabolites regulated microglia homeostasis in mouse models [94].

### 4.1. Underlying Mechanisms behind Microbiota–Gut–Brain Communication

Recent evidence highlights the ability of the gut microbiota to modulate brain functions through the increasingly accepted concept of the microbiota-gut-brain axis [96]. Endocrine and metabolic signals together with neural connections form a bidirectional communication system between the central nervous system (CNS) and ENS, linking emotional and cognitive centers of the brain with peripheral intestinal functions. The underlying mechanisms behind microbiome–gut–brain communication are not yet fully understood but several signaling pathways have been already uncovered. The research findings demonstrated that microbial signals including structural components of bacteria or microbial metabolites can affect distal organs either directly or via neural and hormonal signaling. The systemic inflammation induced by gut dysbiosis can bidirectionally enhance the stress-activated hypothalamic–pituitary–adrenal (HPA) axis via the vagus nerve [97]. The HPA axis plays a key role in coordinating the neuroendocrine stress response and represents an important pathway in the microbiota–gut–brain communication [96].

Microbiota-derived metabolites including SCFA, neurotransmitters, hormones, and immune system modulators contribute to the microbiota–gut–brain communication. SCFA represent the main products of bacterial fermentation of dietary fiber in the intestines [98]. As well as their effect on maintaining the integrity of intestinal membranes and mucus production, the participation of SCFA in microbiota–gut–brain crosstalk through immune, endocrine, vagal, and other humoral pathways has been detected and widely reviewed [99]. SCFA have been shown to cross the blood–brain barrier (BBB) [100], suggesting a possibility of direct SCFA interactions. Moreover, gut bacteria can produce important neurotransmitters including gamma-aminobutyric acid (GABA), acetylcholine, and serotonin. As mentioned, several commensal organisms from *Bifidobacterium* and *Lactobacillus* genera have been reported to produce GABA, a major inhibitory neurotransmitter that is important for a healthy brain and nervous system [101]. It is estimated that up to 90% of the body’s serotonin (5-hydroxytryptamine, 5-HT) is produced in the digestive tract by specialized endocrine enterochromaffin cells (ECs) [102]. Yano et al. demonstrated the key role of the gut microbiota in the regulation of colon and serum 5-HT via interactions with colonic ECs [103]. Besides the involvement of gut-derived serotonin in immune responses, bone development, cardiac functions, and the regulation of enteric motor and secretory reflexes, the connection of serotonin with mood and cognition via the gut-brain axis has been postulated [104]. Norepinephrine is another neurotransmitter known for its role in sensory signal detection, shown to be involved in behavior and cognition as well [105]. Importantly, dopamine serves as a precursor for norepinephrine and epinephrine, and animal studies have suggested the participation of the microbiota in the modulation and host biosynthesis/catabolism of dopamine and norepinephrine [106].

The activation of innate and adaptive immune cells by gut bacteria results in the release of proinflammatory cytokines IL-1β, IL-6, and TNFα into the circulatory system, leading to systemic inflammation [107]. Elevated levels of circulating IL-1β, IL-6, and TNFα were found to be associated with neuropsychiatric disorders in humans [108,109,110]. However, further research is highly warranted to determine whether their findings represent the cause or consequence of neuropsychiatric symptoms.

Currently, data comparing SCFA profiles in cancer survivors and healthy controls are lacking. However, increased fecal concentrations of microbiota-derived propionate and tryptophan metabolites in elite survivors was observed in the mice study. The administration of these metabolites caused long-term radioprotection, the mitigation of hematopoietic and gastrointestinal syndromes, and a reduction in proinflammatory responses [111].

### 4.2. Microbiota–Gut–Brain Axis and Neurological Disorders

Systemic inflammation and neurological dysfunction are linked with gut dysbiosis [112]. According to the preclinical studies, GF mice demonstrated changes in anxiety-like, social and cognitive behavior [113,114]. In short-term antibiotic-treated mice, microbial community disruption confirmed by 16S rDNA sequencing has led to a depletion of bacteria-derived circulating metabolites. Furthermore, antibiotic-induced gut dysbiosis was associated with cognitive impairment and dysregulation of cerebral signaling molecules in treated animals [115]. The repeated administration of two common antibiotics, ampicillin and cefoperazone, to juvenile male BALB/c mice, produced microbial changes linked with a decrease in behavior and cognitive skills. Interestingly, significantly enhanced anxiety- and depressive-like behaviors were observed in ampicillin-treated animals [116].

Since the concept of associations between the gut microbiota and cognitive functioning and psychological well-being is quite novel, large-scale analyses related to the microbial neuroactive potential in humans are still rare. Recently, Valles-Colomer et al. performed a study dealing with the impact of microbiota composition on quality of life in a cohort comprising more than 1050 participants from Belgium’s Flemish Gut Flora Project. A significant correlation between neuroactive metabolites produced by certain bacterial genera and mental well-being has been detected. In particular, butyrate-producing *Faecalibacterium* and *Coprococcus* spp. were positively correlated with a higher quality of life and negatively associated with human depression [117].

Several clinical studies have described the association between altered gut microbial composition and cognitive impairment, pain, and several neuropsychiatric and CNS disorders so far. Microbial analysis of fecal samples from clinically depressed patients and matched non-depressed controls by 16S rRNA sequencing identified the overrepresentation of *Bacteroidales* and underrepresentation of *Lachnospiraceae* associated with depression (*p* = 0.05 and 0.02, respectively) [118]. Jiang et al. identified microbiota signatures specific for depression, showing the increased abundance of Bacteroidetes and Proteobacteria and a decrease in Firmicutes in a group of patients with active major depressive disorder (MDD) [119]. The correlation between lower *Bifidobacterium* and/or *Lactobacillus* counts and depression was observed in fecal samples of MDD patients compared to controls using bacterial rRNA-targeted reverse transcription-quantitative polymerase chain reaction [120]. Recently, a comparative metaproteomics analysis showed statistically significant differences Bacteroidetes, Proteobacteria, Firmicutes, Actinobacteria in MDD patients [121]. Saji et al. demonstrated a relationship between the gut microbiome and dementia in a cross-sectional study conducted on Japanese patients [122]. The gut microbiome of patients with Parkinson’s disease, Alzheimer’s disease, and multiple sclerosis also displayed alterations in gut microbiota composition [123,124,125]. The microbiota composition of mucosal and fecal samples from patients with Parkinson´s disease was enriched by putative, “proinflammatory” Proteobacteria of the genus *Ralstonia*. In addition, a higher abundance of butyrate-producing bacteria from the genera *Blautia*, *Coprococcus*, and *Roseburia*, as well as *Faecalibacterium*, was identified in healthy controls compared to patients [123]. Metagenomic characterization of the gut microbial communities uncovered a lower microbial diversity with a decrease in Firmicutes and *Bifidobacterium* and an increase in Bacteroidetes in the fecal samples of patients with Alzheimer’s disease [124]. Chen et al. described a distinct microbial community profile with an increased abundance of *Pseudomonas*, *Mycoplana*, *Haemophilus*, *Blautia*, and *Dorea* genera in fecal samples from patients with multiple sclerosis compared to healthy controls [125]. Existing human studies of the gut microbiome and particular neurological diseases report distinct results regarding the prevalence and abundance of altered bacterial taxa. Therefore, the study of microbial functioning suggests being more effective than a sole metagenomic approach to determine the clear relationships.

## 5. Chemotherapy-Induced Dysbiosis Associated with Cognitive Impairment, Psychoneurological Symptoms, and Peripheral Neuropathy

Chemotherapeutic drugs affect brain functions, leading to numerous side effects in cognitive functioning. Some chemotherapeutics such as 5-fluorouracil or cyclophosphamide, can directly cross the BBB resulting in oxidative stress, neuroinflammation, or damaging of neurovascular elements. However, many drugs, including paclitaxel and doxorubicin, cannot easily penetrate the the BBB. Hence, indirect mechanisms via peripheral inflammatory mediators suggest inducing neurological changes and impaired cognitive functioning. Chemotherapeutic agents and inflammatory signaling molecules present in the bloodstream can increase the permeability of the blood-brain membrane by a disruption of tight junctions followed by elevated caveolar transcytosis. After crossing the barrier and entering the CNS, the activation of microglia and astrocytes trigger the release of proinflammatory cytokines and ROS. Direct or indirect mechanisms of chemotherapeutics result in neuroinflammation, neuronal damage, and subsequent apoptosis [126].

### 5.1. Gut Microbiome and Chemotherapy-Related Cognitive Impairment

Chemotherapy-related behavioral comorbidities and cognitive impairment might result from altered microbiota–gut–brain communication pathways including neuroinflammation [14,127] and intestinal barrier integrity [128]. Importantly, a recent study in a mouse glioblastoma model suggests that changes in microbiota composition may contribute to a tumor microenvironment remodeling leading to tumor development. As the authors have shown, antibiotic treatment with vancomycin and gentamycin resulted in an early impairment of NK cells, changes in microglia phenotype, and increased growth of intracranial glioma in treated animals. The microbial analysis revealed the lower diversity with the absence of *Prevotellaceae, Rikenellacaea,* and *Helicobacteraceae*, and increased abundance of *Burkholderiales* after the antibiotic administration [129]. Chemotherapy-induced cognitive impairment and psychological distress belong to the most frequent late effects in survivors including the patients with brain tumors such as glioma, glioblastoma, and primary central nervous system lymphoma [130].

**Figure 1:**
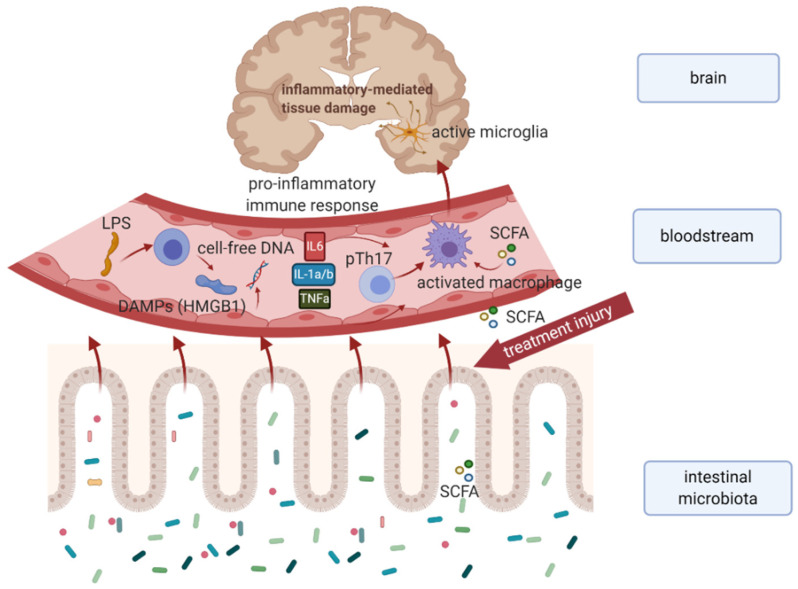
Hypothetical model explaining the immune-related mechanism of cancer treatment-induced cognitive dysfunction in survivors. Chemo- or radiotherapy-related dysbiosis, and intestinal barrier disruption result in an increased level of microbiota-derived metabolites (e.g., SCFA), bacterial LPS, and DAMPs, as well as cell-free DNA in systemic circulation leading to proinflammatory immune response. Subsequent activation of microglial cells results in neuroinflammation and neuronal apoptosis associated with cognitive impairment. *Abbreviations:* DAMPs, damage-associated molecular patterns; HMGB 1, high-mobility group box 1; IL-1a/b, interleukin 1a, and 1b; IL6, interleukin 6; LPS, intestinal microbiota associated lipopolysaccharide; SCFA, short-chain fatty acids produced by intestinal microbiota; TNFa, tumor necrosis factor-alpha.

Recently, the existence of a possible relationship between the chemotherapy-modified microbiota-gut-brain axis and impaired cognitive functioning in cancer survivors represents an emerging field of this research area. According to the available research findings, an immune-related pathway of cancer treatment-induced cognitive dysfunction via the microbiota–gut–brain axis might represent a possible mechanism (Figure 1).

Disruption of intestinal microbiota and subsequent dysbiosis result in the gut pathogenic microbiome, and the generation of lipopolysaccharides (LPS) derived from the cell wall of Gram-negative bacteria. This bacterial endotoxin may disrupt intestinal integrity and LPS efflux from the gut contributes to neuroinflammation and oxidative stress followed by glial activation in the hippocampus [131,132]. It has been reported that LPS induces inflammation by binding to microglial toll-like receptors (TLRs) and evoking M1 microglial activation associated with a reduction in neurogenesis [133]. In particular, the binding of LPS to microglial TLR4 suggests activation of the inflammatory cascade by NF-κB and proinflammatory cytokines (TNF-α, IL-1β, and COX2), resulting in elevated neuroinflammation [134].

Chemotherapy-induced dysbiosis leads to a dysregulation of the microbiota–gut–brain axis at distinct stages. In a recent preclinical study, a correlation between chemotherapy-induced changes in the intestinal microflora and neuroinflammatory changes in the brain was observed in mice after paclitaxel treatment. Results confirmed that elevated circulating cytokine levels and neuroinflammation were associated with cognitive impairment, anxiety, and mood disorders. Moreover, enhanced neuroinflammation and whole-body immune response were detected after the treatment. The intestinal microbiome of treated animals changed towards the reduction in butyrate-producing bacteria (e.g., *Lachnospiraceae*) compared to the control group. Taken together, the results showed chemotherapy-induced anorexia, slowed growth, cognitive impairment, and an increase in central and peripheral inflammatory processes, as well as increased endotoxin levels in the bloodstream. Hence, the modulation of the intestinal microbiota might represent a potential therapeutic strategy not only for reducing the gastrointestinal side effects of chemotherapy but also for mitigating the impact on neurological functions [14].

The results from available clinical studies indicated that cancer treatment-related psychoneurological symptoms and toxicities can be mediated by the microbiota–gut–brain axis. A cross-sectional study by Okubo et al. provided the first evidence that chemotherapy-induced changes in gut microbiota influenced the fear of cancer recurrence (FCR) among breast cancer survivors. The metagenomic analysis found a link between a higher relative abundance of *Bacteroides* and higher FCR. On the other hand, a lower FCR was associated with a higher relative abundance of *Lachnospiraceae* and *Ruminococcus*. In addition, lower bacterial diversity was significantly associated with higher FCR [31]. The link between the gut microbiome changes and alterations in psychosocial factors including anxiety, depression, fatigue, sleep quality, and cardiorespiratory fitness was found in a proof-of-concept study on a cohort of 12 breast cancer survivors. Several bacterial taxa as *Bacteroides*, *Roseburia*, and *Prevotella* were significantly associated with changes in psychosocial symptoms. The change in fatigue interference correlated with the frequency of genera *Faecalibacterium* and *Prevotella* whereas the change in anxiety was associated with the frequency of genera *Coprococcus* and *Bacteroides* [32]. Very recently, Bai et al. demonstrated pre- and post-radiotherapy correlations between microbial diversity and psychoneurological symptom (PNS) cluster in a pilot study comprising 13 patients with head and neck cancers [30]. PNS cluster has been previously defined as a set of symptoms including pain, fatigue, sleep disturbance, depressive symptoms, and cognitive dysfunction [135], reliably associated with reduced quality of life. Microbial analysis of stool samples from cancer patients showed higher abundances of *Ruminiclostridium9*, *Tyzzerella*, *Eubacterium_fissicatena*, and *DTU089* in patients with the high PNS cluster. On the other hand, patients with the low PNS cluster displayed higher abundances of *Lactococcus*, *Phascolarctobacterium*, and *Desulfovibrio*. Importantly, significant differences in both glycan and vitamin metabolism between the high and low PNS clusters pre- and post-radiotherapeutic treatment were observed [30].

Currently, several ongoing large-scale clinical studies on young adult cancer survivors after chemotherapeutic treatment [136] and pediatric patients with solid tumors [137] aim to determine the role of the gut microbiome in treatment-induced short-term and long-term side effects.

### 5.2. Gut Microbiome and Chemotherapy-Induced Peripheral Neuropathy

A key role of the intestinal microbiota in the development of inflammatory pain has been detected by measuring the hypernociceptive responses in germ-free and conventional mice [138]. Recent evidence supports the significant impact of the gut microbiome on neuropathic pain providing the potential for novel therapeutic strategies [139]. CIPN and the gut microbiome may be linked via the immune-nervous-endocrine axis [140]. CIPN, characterized by pain, muscle weakness, numbness, burning, or tingling, is the long-lasting toxic side effect of cancer treatment with taxanes, platinum compounds, and other commonly used anti-cancer drugs [141]. Neurotoxic effects of chemotherapeutics leading to the production of ROS, and activation of pain receptors, together with neuroinflammation represent the possible mechanisms underlying CIPN. The results showed that cognitive impairment and distinct psychological disorders are often linked to CIPN. Interestingly, a striking association between gut microbiota and CIPN has been uncovered, showing reduced oxaliplatin-induced mechanical hyperalgesia in GF mice, or animals with temporarily eradicated gut bacteria by antibiotics [142]. Accordingly, Ramakrishna et al. observed a crucial role of gut bacteria in paclitaxel-induced pain sensitivity and resistance when comparing the microbiota composition of C57BL/6 (B6) and 129SvEv (129) mice. In their study, microglia were found to be causally involved in paclitaxel-induced pain symptoms, and the possible interplay of several bacterial taxa was identified. From initial microbiota (before paclitaxel administration), *Lactobacillus intestinalis* and *Eubacterium siraeum* were suggested to be inhibitors of the pain phenotype. In addition, the pain inhibiting phenotype after paclitaxel administration was supposed to be driven by the members of *Porphyromonadaceae*. Since paclitaxel decreased the abundance of *Akkermansia muciniphila*, altered brain functions might have resulted from changes in communication via the microbiota-gut-brain axis [128]. Recently, an association between increased levels of circulating butyrate and neuropathic pain improvement following fecal microbiota transplantation (FMT) in obese mice has highlighted the novel approaches for neuropathy prevention, or pain relief [143].

## 6. The Relationship between the Gut Microbiota and Cardiovascular Toxicity

Cardiovascular complications such as heart failure, myocardial ischemia, hypertension, thromboembolism, and arrhythmias are among the most life-threatening late toxicities of platinum-based chemotherapy and radiotherapy in cancer survivors. Importantly, high cardiovascular toxicity is a reason for cancer treatment interruption. The cardiotoxic effects of cancer treatments have been studied for the last 20 years leading to the identification of some important key players in downstream pathways. The most relevant and studied forms of cardiac dysfunctions represent apoptosis and necrosis of cardiomyocytes, and abnormalities in myocardial energetics due to anthracyclines, mainly doxorubicin [144,145]. Moreover, in vitro studies showed the impact of anthracyclins on cultured cardiac endothelial cells [146] and fibroblasts as well [147,148].

Different mechanisms linking antineoplastic drugs with cancer treatment-related cardiotoxicity, and the existing link between intestinal microbiota composition and cardiovascular diseases have been described, including the development of atherosclerosis and heart failure [149]. Several studies have shown significantly decreased microbiota diversity and altered intestinal membrane permeability in patients with heart failure compared to controls. Heart failure with a reduced ejection fraction leads to reduced ejection volume, intestinal hypoperfusion, and venous overload, followed by local ischemia and the formation of intestinal wall edema. Subsequent changes in the gut barrier and microbiota composition trigger bacterial translocation and inflammatory response. Moreover, the progression of heart failure is associated with the presence of LPS and metabolites related to cardiovascular diseases in the bloodstream [150]. Chronic activation of the sympathetic nervous system increases cardiomyocyte apoptosis and the release of vascular growth hormone, which contributes to vasoconstriction and atherosclerosis [151].

Chemotherapy-induced intestinal barrier disruption results in the leakage of endotoxin into the bloodstream. Bacterial LPS has been found to promote the release of inflammatory cytokines in cardiovascular diseases [152], and circulating endotoxin was elevated in patients with heart failure [153]. The mammalian endotoxin sensor TLR4 has been shown to be associated with doxorubicin-induced cardiopathy, since no heart failure was observed in TLR4-knockout mice after doxorubicin treatment [154]. Accordingly, Wang et al. reported the involvement of TLR4 in doxorubicin-induced damage in the heart, kidney, liver, and intestine. The research on mouse models suggested that depletion of gut microflora or inhibition of TLR signaling might represent an effective approach for alleviating doxorubicin toxicity, and possible implementation for other chemotherapeutics was also being considered [155]. However, other microbial polysaccharides, and TLR2 and TLR9–mediated nucleic acids-inducing inflammation were also linked to doxorubicin toxicity [156]. Recently, doxorubicin-induced cardiotoxicity was prevented by glabridin (GLA)-mediated modulation of microbial dysbiosis and colonic macrophage polarization in mice. The underlying mechanisms might be associated with decresed LPS and increased butyrate observed in the feces and peripheral blood [157].

Importantly, a significant reduction in SCFA-producing bacteria in patients with heart failure suggested their cardioprotective effect. SCFAs are likely to mediate post-infarction repair of the heart by infiltrating CX3CR1-positive monocytes into the peri-infarct zone. Moreover, butyrate´s anti-inflammatory effect and induction of Foxp3-positive Treg cells led to a suppression of Th17 formation. In addition, SCFAs help to maintain the intestinal barrier integrity through hypoxia-inducing factor expression. The ability of propionate to modulate blood pressure has also been demonstrated in mouse models [158].

In conclusion, limited knowledge exists regarding the interplay among the microbiome, vessel damage, and heart failure in cancer patients after treatment. Microbiome studies on a large cohort of cancer survivors may lead to the identification of patients at cardiovascular risk who could profit from a more personalized microbiota-mediated approach. Moreover, a better understanding of the association between the gut microbiome and cancer treatment-related cardiotoxicity might bring the possibility to reduce the risk of this serious and lethal adverse effect. The recent findings suggest that gut microbiota modification concerning SCFA-producing bacteria could prevent cardiovascular disease and thus represent a potential therapeutic strategy [159].

## 7. Gut Microbiota Modulation as an Emerging Trend in Cancer Survivors

Interventions and supportive care for treatment-induced long-term effects remain an emerging area of research in cancer survivors [160]. Due to the lack of preventive measures and approved pharmacological agents, different possibilities in preventing or mitigating the late toxicities need to be assessed. Targeting the microbiota-gut-brain axis in cancer survivors might represent a new potential trend being in its infancy to date. Gut microbiota disruption after chemo- and radiotherapy can be recovered by several mechanisms including administration of probiotics and/or prebiotics, and FMT. Interestingly, the relationship between diet, physical activity, and gut microbiome appears to be another potential tool in cancer survivors. However, most of the data dealing with neuro- and cardioprotective effects of microbiota modulation come from preclinical and non-cancer patient clinical studies, and further evaluations of cancer patients are highly warranted.

### 7.1. Neuro- and Cardioprotective Effect of Probiotics

In cancer patients, the administration of probiotics mainly aims to alleviate the adverse effects of chemo- and radiotherapy and reduce gastrointestinal toxicity while increasing bacterial diversity. Interestingly, a survey study comprising 499 cancer patients documented a probiotic consumption in 28.5% of all participants [161]. Several studies focusing on the pre- and post-treatment probiotic supplementation reported improved immune responses and the reduction in infectious complications in patients with a different spectrum of malignancies [162,163,164]. Probiotic bacteria produce antimicrobials, compete with pathogens for nutrients, or adhere to intestinal epithelial cells, and physically block the adhesion of pathogens resulting in high colonization resistance [165].

A limited number of clinical trials concerning probiotic use to ameliorate the chemotherapy-related side effects on behavioral comorbidities or cognitive impairment have been conducted so far. Lee et al. showed that *Lactobacillus rhamnosus* and *Lactobacillus acidophilus* were able to reduce the symptoms of depression, anxiety, and fatigue in colorectal cancer survivors [166]. Recently, a randomized double-blind and placebo-controlled trial comprising 120 elderly patients following elective orthopedic or colorectal cancer surgery found an association between the perioperative application of oral probiotics and postoperative reduction in cognitive impairment. In addition, increased microbiota diversity and decrease plasma IL-6 and cortisol levels were observed in the group of probiotic patients suggesting a possible mechanism via reducing the peripheral inflammation, and the stress response [167]. Interestingly, the probiotic intervention was found to reduce the clinical anxiety before surgery by the suppression of serum corticotropin-releasing factor levels and avoiding the increase in heartbeat among patients with laryngeal cancer [168].

Neuroprotective effects of probiotics have been detected in numerous experimental models and clinical trials dealing with behavioral dysfunctions and neurodegenerative disorders. Probiotic metabolites such as SCFA play a role in maintaining the BBB integrity through the increased expression of claudin and occludin in the membrane. Moreover, the production of tryptophan metabolites might block proinflammatory NFk-B and VEGF-B, the activation of astrocytes, and microglial cells within the brain [169]. According to the findings from mouse models, long-term probiotic administration reduced anxiety and depression, normalized the immune response, caused changes in GABA production, diminished oxidative stress markers in the brain, enhanced the activities of antioxidant enzymes, preserved neuronal synaptic plasticity, and restored basal noradrenaline levels in the brainstem [170,171,172,173]. A positive link between *Bifidobacterium longum* 1714 consumption and stress reduction as well as improved memory was indicated in a clinical study comprising male healthy participants [174]. Accordingly, another study on human volunteers showed beneficial effects of the oral intake of *Lactobacillus helveticus* R0052 and *Bifidobacterium longum* R0175 on anxiety and depression-related behaviors [175]. Wallace et al. reviewed the effects of probiotics on depressive symptoms and provided a list of clinical trials dealing with probiotic use for depression and anxiety [176]. In a recent meta-analysis of 19 double-blind, randomized, placebo-controlled trials, Goh et al. confirmed the beneficial effect of probiotics on depressive symptoms in patients with MDD [177].

Besides neuroprotective effects, cardioprotective effects of probiotics have also been reported and extensively reviewed [178,179]. In particular, mouse and rat models have demonstrated reduced cardiomyocyte apoptosis, a protective effect of myocardial damage, improved cardiac function, and survival in animals after exposure to *Lactobacillus* spp. [180,181]. According to the findings, the administration of a probiotic *Lactobacillus rhamnosus* GR-1 has attenuated post-infarction remodeling and heart failure in rats subjected to sustained coronary artery ligation [182]. A cardioprotective effect against heart ischemic injury through the attenuation of TNF-α and oxidative stress was observed in a rat myocardial infarction model after receiving the combination of four viable probiotic bacteria strains *Bifidobacterium breve*, *Lactobacillus casei*, *Lactobacillus bulgaricus*, and *Lactobacillus acidophilus* [183]. Importantly, probiotic consumption in patients with heart failure has led to an improvement in disease-related parameters [184]. In accordance, the reduced risk of cardiovascular diseases in patients with metabolic syndrome was found after probiotic administration [185].

### 7.2. Fecal Microbiota Transplantation and Improvements in Neurologic Functions and Cancer Treatment Efficacy

FMT represents the transfer of intestinal microbiota from a healthy donor into the patient’s intestine. Currently, FMT is predominantly used in the treatment of severe and life-threatening intestinal inflammation caused by *Clostridium* spp. where antibiotic treatment fails. Nevertheless, FMT modulation was associated with improvements in neurologic functions, possibly along the microbiota–gut–brain axis.

In particular, a reduction in cognitive deficits, a decrease in TNF-induced neuroinflammation, an increase in serotonin levels, as well as improvement in motor skills in mouse models of Alzheimer’s and Parkinson’s disease were reported [186,187]. Bercik et al. demonstrated the changes in brain chemistry and behavior after microbiota disruption in healthy mice. According to their results, adoptive transfer experiments with cecal bacteria reported the altered exploratory behavior of GF mice after colonization with microbiota from different mouse strains [188]. Translational studies concerning the transplantation of patients´ gut microbiota to GF or microbiota-deficient rodents documented alterations in several neurobehavioral features. Specifically, FMT from a subgroup of patients with MDD to microbiota-depleted rats induced a depression-like phenotype, including anhedonia and anxiety-like behaviors in the recipient animals not observed in recipients of FMT from healthy control individuals. In addition, the results showed significant differences in the relative abundance of Firmicutes, Actinobacteria, and Bacteroidetes in gut microbiota compositions between depressed patients and healthy controls [189,190].

Preclinical and clinical findings suggest an increasing trend of FMT in the management of cancer patients, and its use in oncology is encouraging. Data from colorectal cancer-bearing mice showed that FMT safely alleviated FOLFOX (5-fluorouracil, leucovorin, and oxaliplatin)-induced intestinal mucositis [191]. Importantly, the functional impact of microbiota on cancer treatment efficacy has been documented, showing improved response to anti-PDL-1 immunotherapy in antibiotic-treated or GF mice bearing tumors after FMT from patients responding to cancer treatment compared to FMT from non-responders [24,25]. Metagenomic analysis of fecal samples collected from mice treated with FMT from responding patients showed a high diversity and abundance of *Ruminococcaceae*/*Faecalibacterium* [24]. Accordingly, Matson et al. suggested that the gut microbiome might have a mechanistic impact on antitumor immunity as “reconstitution of germ-free mice with fecal material from responding patients led to improved tumor control, augmented T cell responses, and greater efficacy of anti-PD-L1 therapy” [79].

Several clinical studies concerning the use of FMT in cancer patients receiving high-dose chemotherapy regimens prior to hematopoietic stem cell transplantation showed improved patient outcomes regarding the decrease in infectious complications and graft-versus-host-disease. However, no correlations with cognitive or cardiovascular functioning have been monitored [192,193,194,195,196,197]. Currently, several clinical trials concerning the impact of FMT on the increasing cancer treatment efficacy are ongoing [198]. According to the ClinicalTrials.gov database (accessed on 13 December 2020), the clinical trials NCT03341143 and NCT03353402 are assessing the effect of a fecal microbiota transplant from patients who responded to immunotherapy by PD-1 blockade to non-responding metastatic melanoma patients who failed immunotherapy. Furthermore, a clinical trial NCT04116775 addresses the anticancer effect of FMT from responders to pembrolizumab into non-responders in a cohort of patients with metastatic castration-resistant prostate cancer (http://clinicaltrials.gov/, accessed on 13 December 2020).

In conclusion, FMT might become a potential novel approach in the treatment of chemotherapy-related side effects on brain functions associated with intestinal microbiota disruption. However, further preclinical research focusing on the safety and efficacy of FMT is needed to increase the potential of application in the cancer population. Moreover, a documented case of FMT-related death in a cancer patient reinforces the need for the more detailed and precise screening of donors for the presence of multi-resistant bacterial pathogens [199].

### 7.3. The Possible Impact of Diet and Physical Activity on the Gut Microbiome in Cancer Survivors

Diet represents an important factor influencing intestinal microbiota homeostasis [200]. Malnutrition and changes in diet composition have been reported in cancer patients [201] and the potential link between the gut microbiome and psychoneurological symptoms via microbiota–gut–brain communication has been proposed. Although the studies of diet–microbiota–cancer interactions are still very scarce, the impacts of high-quality diet on PNS cluster and quality of life in breast cancer survivors have been intensively studied and widely reviewed [202]. A large cross-sectional study on breast cancer survivors (*n* = 746) revealed that patients with a high-quality diet, defined as a diet rich in fruits, vegetables, whole grains, and polyunsaturated fatty acids and low in added sugar, had lower levels of chronic inflammation compared to survivors with the poorest diet quality [203]. A direct association of diet quality with subsequent mental and physical functioning was found in breast cancer survivors (*n* = 714) who participated in the Health, Eating, Activity, and Lifestyle (HEAL) study [204]. Recently, Huang et al. demonstrated higher post-therapy cognitive scores regarding verbal fluency and improvements in delayed memory in breast cancer survivors with a higher vegetable intake, tea-drinking, and fish oil supplementation [205].

Animal models, as well as clinical studies on elite athletes and healthy subjects, indicate the positive effect of physical activity on gut microbiota diversity and the production of beneficial metabolites [206,207]. Importantly, several clinical trials concerning cancer survivors have reported an association between exercise and clinically meaningful improvements in quality of life [208] and mortality [209]. However, the relationship between exercise and gut microbiota in cancer survivors requires further investigation. Currently, a single-blinded, two-armed, randomized, controlled trial aims to examine whether exercise favorably alters gut microbiota in the patients receiving androgen deprivation therapy for prostate cancer [210]. The results of ongoing clinical trials concerning the link between diet, physical activity, and microbiome alterations in cancer survivors (Table 1) may bring some interesting contributions to this field.

## 8. Conclusions and Future Directions

Due to the lack of specificity of most chemotherapeutics to target only malignant cells, cancer patients experience numerous acute and long-term side effects throughout the body, including gastrointestinal toxicity, cognitive and behavioral impairment, and cardiotoxicity. The increasing number of long-term survivors highlights the need for the elucidation of the underlying mechanisms in chemotherapy-induced late effects. Recently, animal models and clinical studies have uncovered the significant association between changes in the intestinal microbiota and treatment-related comorbidities. Therefore, the emerging role of the gut microbiome in late effects among cancer survivors is gaining more attention, and prospective, longitudinal clinical studies in chemotherapy-treated patients represent a major challenge.

The microbiota actively interacts with the host and we are just at the beginning of deciphering the exact signals of communication. The enormous microbial diversity among cancer patients suggests that the most efficient therapies may be directed at the unique microbiota composition rather than individual bacterial strains. A deep understanding of the microbiota–gut–brain axis in cancer and elucidating the impact of an altered intestinal microbiome on immune, metabolic, psychological, and cognitive pathways is crucial for improving the physical and psychosocial health of survivors. Microbiota modulation by probiotics and prebiotics or FMT might represent an emerging trend in cancer survivors. However, current clinical trials concerning the neuro- and cardioprotective effects of probiotics or FMT are still rare, comprising mainly non-oncologic patients. Moreover, limitations in sample size, discrepancies in combinations of probiotic strains, and the length of treatment should be taken into account when considering the efficacy and safety of probiotic use. In the future, randomized controlled clinical trials on a large cohort of cancer survivors are highly warranted and could bring new perspectives for microbiota-mediated interventions to prevent or mitigate the chemotherapy-induced long-term effects.

## Figures and Tables

**Table 1:** Cancer survivorship and the microbiome. The table summarizes the list of ongoing and completed clinical trials dealing with the impact of the microbiome on cancer survivorship (according to http://clinicaltrials.gov/, accessed on 13 December 2020).

Study	Study Design	Disease	Purpose	Patients (*n*)	Intervention	Study Status
NCT03760653	A prospective, randomized double-blind study	Breast cancer survivors	To determine the effects of physical exercise together with the supplementation of a probiotic on the gut microbiota balance, the gut immune system, and quality of life (intended as functional and muscular capacity, physical qualities, and emotional state) in breast cancer survivors.	30	Physical exercise and probiotic group vs. probiotic group vs. placebo	Suspended (the project abandonment by the research who recruited the patients)
NCT04088708	A prospective, randomized, single-blind study	Breast cancer survivors	To determine exercise effects on the number, distribution, and types of bacteria in the gut of breast cancer survivors.	126	Aerobic exercise training vs. attention control	Ongoing
NCT02843425	A prospective, randomized, open-label, cross-over study	Colorectal cancer survivors	To determine the effect of pre-cooked beans on the levels of healthy bacteria in the digestive system and reduction in obesity effect on cancer risk.	80	Regular diet + beans, then regular diet—beans vs. regular diet—beans, then regular diet + beans	Active, not recruiting
NCT04097353	A prospective, randomized, open-label study	Pediatric cancer survivors	To examine the efficacy of Harvesting Hope for Kids (HH4K), a biobehavioral intervention delivered in the context of a university-based, cancer survivor garden to increase produce intake and physical activity in survivors and caregivers including changes in microbiome composition.	75	Harvesting Hope for Kids (HH4K) vs. Surviving Strong for Kids (SS4K)	Enrolling by invitation
NCT03781778	A prospective, randomized, double-blind study	Stage I-III colorectal cancer survivors	To test the effect of the consumption of foods made with resistant starch compared to foods made with corn starch on biomarkers that may be related to colorectal cancer progression in stage I-III colorectal cancer survivors.	NA	Resistant starch foods vs. foods with regular corn starch	Terminated (funding expiration)
NCT04499950	A non-randomized, single-arm, phase II study	Breast cancer survivors	To determine the effects of pharmacotherapy and a remote behavioral weight loss intervention on weight loss in breast cancer survivors who are overweight or obese and the impact of successful weight loss on serum biomarkers and the gut microbiome.	55	POWER-remote behavioral weight loss intervention	Not yet recruiting
NCT01929122	A prospective, randomized, single-blind study	Colorectal cancer survivors	To explore the effects of cooked navy bean powder or rice bran consumption on the stool microbiome and metabolome of colorectal cancer survivors and healthy adults.	29	Cooked navy bean powder vs. rice bran vs. placebo	Completed

**Table 2:** Summary of known cardiovascular and neurocognitive toxicity and the alterations of intestinal microbiota as a result of treatment with chemotherapy.

Chemotherapy Agent/Agent-Based Regimen	Cardiovascular Toxicity	Neurocognitive Toxicity	Known Effects on Gut and the Microbiome
Anthracyclines	Congestive heart failure, left ventricular dysfunction, arrhythmia, cardiomyopathies [80]	Cognitive impairment [5], peripheral neuropathy [81]	Increased intestinal permeability [73]
Cyclophosphamide/Ifosfamide	Congestive heart failure, left ventricular systolic dysfunction [82]	Cognitive impairment [5], peripheral neuropathy [81]	Translocation of Gram-positive bacteria into mesenteric lymph nodes and spleen [27], disrupted intestinal barrier integrity [74]
Taxanes	Arrhythmias, cardiac ischemia, left ventricular dysfunction [83,84]	Cognitive impairment [6], peripheral neuropathy [81,85]	Decreased abundance of *Akkermansia muciniphila*, disrupted intestinal barrier integrity [86]
Etoposide	Not significant	Occasional peripheral neuropathy [81]	Increased intestinal permeability [73]
Cisplatin/Carboplatin	Coronary artery disease, hypertension, myocardial infarction, Raynaud phenomenon [4]	Cognitive impairment [6], peripheral neuropathy, paresthesia, ototoxicity [4,85]	Dysbiosis, antimicrobial effect on *Bacillus*, *Escherichia coli*, disruption of intestinal mucosa [87]
Cytarabine	Pericarditis [88]	Neurocognitive deficits [89]	Unknown
5-Fluorouracil, Capecitabine, Gemcitabine	Coronary spasms, ischemia [83]	Senzory neuropathy, paresthesia after gemcitabine [81], cognitive impairment [86]	Intestinal mucosal damage, lower abundance of Firmicutes, increase in Bacteroidetes, Actinobacteria and Verucomicrobia [90]
Methotrexate	Not significant	Cognitive deficits, impaired executive functions [91]	Reduction in *Bifidobacterium*, *Lactobacillus*, *Escherichia coli* [75], mucosal barrier disruption [73]
Myeloablative chemotherapy (Carmustine, Etoposide, Aracytine, Melphalan)	Hypertension, diabetes, left ventricular dysfunction, arrhythmia, stroke, myocardial infarction, heart failure [44]	Adverse psychosocial effects, mental health disorders, cognitive impairment [89]	A decrease in Firmicutes and Actinobacteria and an increase in the abundance of Proteobacteria [76]
